# A survey on UK researchers’ views regarding their experiences with the de-identification, anonymisation, release methods and re-identification risk estimation for clinical trial datasets

**DOI:** 10.1177/17407745241259086

**Published:** 2024-06-19

**Authors:** Aryelly Rodriguez, Steff C Lewis, Sandra Eldridge, Tracy Jackson, Christopher J Weir

**Affiliations:** 1 Edinburgh Clinical Trials Unit, Usher Institute, The University of Edinburgh, Edinburgh, UK; 2 Pragmatic Clinical Trials Unit, Blizard Institute, Barts and The London School of Medicine and Dentistry, Queen Mary University of London, London UK; 3 Asthma UK Centre for Applied Research, Usher Institute, The University of Edinburgh, Edinburgh, UK

**Keywords:** Clinical trials, data anonymisation, re-identification, de-identification, data sharing, re-identification risk

## Abstract

**Background:**

There are increasing pressures for anonymised datasets from clinical trials to be shared across the scientific community. However, there is no standardised set of recommendations on how to anonymise and prepare clinical trial datasets for sharing, while an ever-increasing number of anonymised datasets are becoming available for secondary research. Our aim was to explore the current views and experiences of researchers in the United Kingdom about de-identification, anonymisation, release methods and re-identification risk estimation for clinical trial datasets.

**Methods:**

We used an online exploratory cross-sectional descriptive survey that consisted of both open-ended and closed questions.

**Results:**

We had 38 responses to invitation from June 2022 to October 2022. However, 35 participants (92%) used internal documentation and published guidance to de-identify/anonymise clinical trial datasets. De-identification, followed by anonymisation and then fulfilling data holders’ requirements before access was granted (controlled access), was the most common process for releasing the datasets as reported by 18 (47%) participants. However, 11 participants (29%) had previous knowledge of re-identification risk estimation, but they did not use any of the methodologies. Experiences in the process of de-identifying/anonymising the datasets and maintaining such datasets were mostly negative, and the main reported issues were lack of resources, guidance, and training.

**Conclusion:**

The majority of responders reported using documented processes for de-identification and anonymisation. However, our survey results clearly indicate that there are still gaps in the areas of guidance, resources and training to fulfil sharing requests of de-identified/anonymised datasets, and that re-identification risk estimation is an underdeveloped area.

## Background

There is now a strong drive, particularly from publishers and funders, to encourage the release of relevant anonymised trial data sets.^1^ Therefore, data sharing has become an essential activity to disseminate current research, to enable new investigations and to maximise the scientific endeavour.^2,3^ Currently, many anonymised datasets are made publicly available for secondary research via open or controlled access.^4–6^ Anonymisation of data is complex, and its full implementation could mean that the detail necessary to appropriately analyse the data is lost.^7^ There is therefore a balance between wanting to de-risk a dataset prior to sharing, against wanting it to be sufficiently detailed to answer valid research questions, and to allow researchers to repeat the original published analysis. In addition, we are currently investigating re-identification risk scores across a range of clinical trial datasets.^8^ Re-identification risk scores, as described by in the work by El Emam,^9^ are derived from three equations that use information in the anonymised dataset. They are currently used for routinely collected health records and only generate numerical values. These scores do not aim to re-identify individuals in the datasets and could potentially be applicable to clinical trial datasets. Therefore, we explored UK researchers’ views regarding their experiences with the creation and release of de-identified/anonymised clinical trial datasets, the generation and use of re-identification risk scores, and their views about wider aspects of re-identification risks. Humphreys et al.^10^ covered wider aspects of data sharing in clinical trials while our study focuses on clinical trial datasets that have been anonymised/de-identified.

### Why it is important to do this study?

Knowing what is working and what is not regarding the creation and release of de-identified/anonymised clinical trial datasets, and determining if re-identification risk scores are already in use, from UK clinical trials researchers, will help identify areas for improvements and future research.

### Objective

This study aimed to explore the clinical trial researchers’ views on their experiences with the creation and release of de-identified/anonymised clinical trial datasets, and the generation and use of re-identification risk scores, and the wider aspects of re-identification risks.

## Methods

A full protocol (in the supplementary material, Additional File 1) and a survey instrument (in Additional File 2) were finalised on 28 April 2022. A non-personal invitation letter was generated to describe the study to potential participants (Additional File 5), before they fully engaged with the survey. The invitation letter and the first part of the survey emphasised the voluntary nature of participation, the protection and handling of personal data, and confidentiality. Consent was obtained from the respondents to participate in the survey, and they were assured they could stop and dropout at any time during the study without any consequences.

### Survey design

The ‘checklist of questions for designing a survey study plan’ by Creswell and Creswell^11^ was followed for the development of this study (see Additional File 3). We used an online exploratory cross-sectional descriptive survey^11,12^ that consists of both open-ended and closed questions for data collection. This allowed us to gather information to better describe actual experiences regarding the investigated topic. The open-ended questions were especially important because of the lack of previous reporting on researchers’ views and experiences.

The survey was in English. Most of the closed questions had mutually exclusive choices, with a smaller number allowing for multiple answers.^13,14^ Where applicable, closed questions, had an ‘other’ (free text) option added to allow participants to provide an answer that was not available for selection.^13^ Five-point response scales were used for questions assessing frequency (*always, often, sometimes, rarely, never*).

The survey was structured in five parts:

- Consent and eligibility check.
- Section 1. Researchers’ work background details (current position, years of experience in current position and general place of work)
- Section 2 Researchers’ experiences with the creation and release of de-identified/ anonymised clinical trial datasets
- Section 3. Researchers’ awareness, knowledge and use regarding the generation of re-identification risk scores as described in the work by El Emam^9^
- Section 4. Researchers’ views about wider aspects of re-identification risks

Where applicable, we also provided short explanations of the concepts used in the survey at the beginning of the relevant section to avoid ambiguity and confusion, as follows:

- De-identification refers to the removal of all personal health information and all other indirect identifiers which could lead to the identification of an individual. The most common de-identification methods are HIPPA (US Health Insurance Portability and Accountability Act of 1996) Safe harbour,^15^ in which 18 identifiers are removed from the datasets and Hrynaszkiewicz et al.^16^ with an enhanced removal of potential identifiers which are commonly present in clinical trial datasets.
- Anonymisation is when a dataset has been de-identified and then subsequent data manipulation/steps have been taken to further protect the dataset, for example, if a privacy model has been applied (e.g. k-anonymity)^17^ or the link with the original non-anonymised dataset has been destroyed and this action cannot be reversed.
- Data release under controlled access: Datasets that can only be accessed if permission is granted by the data holders via their internal procedures.
- Data release under open access: Datasets that can be accessed without any or minimal restrictions imposed by the data holders.
- Re-identification risk scores^9^ are defined as the estimated probabilities of any given individual being re-identified from an anonymised/de-identified dataset. The re-identification risk score depends on the variables available in the dataset, the number of observations in the dataset and on the strategy used to attack the dataset (prosecutor or journalist scenario).
- Prosecutor scenario^9^ is when the adversary knows that a target individual (for whom identifiers are known) is in the publicly available dataset (released anonymised and de-identified).
- Journalist scenario^9^ is when the adversary sets out to identify any individual from the publicly available dataset just to prove that it can be done using another dataset for ‘matching’ with the publicly available dataset.

The final version of the survey is presented in Additional File 2 of this study.

The survey was designed to follow the layout presented in Additional File 4. Therefore, a single participant (after the eligibility criteria has been met) answered between 17 and 22 questions out of the proposed 24 questions, as some answers determined the relevance of the next question.

The survey was piloted using a selection of University of Edinburgh personnel with experience in the processes of de-identification/anonymisation, release/maintenance and re-identification risk assessment of clinical trial datasets. It was then finalised and sent to the intended participants.

### Study population

Inclusion/Exclusion criteria. Clinical trial researchers based in the United Kingdom with experience in executing/overseeing any of the processes of de-identification/anonymisation, release/maintenance and re-identification risk assessment of clinical trial datasets to prepare them for secondary research.

### Sampling and recruitment

There was no formal sample size or stratification of the surveyed researchers as this is an exploratory study. Therefore, we used convenience non-probability sampling^12,18,19^ by providing a Microsoft (MS) Form^20^ link or quick response (QR) code with an invitation letter (email or printout) (see Additional File 5) to the following:

- All 52 Clinical Trial Units^21^ (CTUs, which are the specialised units that design, execute, analyse and publish clinical trials) registered in the UK Clinical Research Collaboration (UKCRC) network.^22^ We emailed all UK fully or provisionally registered CTUs (used list is in Additional File 6).
- The data transparency group at the Global Healthcare Data Science Community (Pharmaceutical Users Software Exchange)^23^ (Contacted via email, population size unknown).
- Allstat@JISCMAIL.AC.UK, a statistics email discussion list for the UK Education and Research communities^24^ (Contacted via email, population size unknown).
- Participants at the Sixth International Clinical Trials Methodology Conference (ICTMC) (3–6 October 2022; Special event) (Contact via leaflet and a QR code in an oral presentation, Population size unknown).

The aim was to obtain as many responses as possible while the main survey was active (around 5 weeks) to maximise the range of experiences. We estimated the population to be heterogeneous, so a minimum of between 12 and 30 surveys was required^18^ to reach data saturation^25^ and reflect a wide range of views.

### Data collection and analysis

This survey did not collect any personal data from the clinical trial researchers, and after extraction, all open questions were carefully checked to make sure their coding did not contain any identifiable information. Only A.R. was able to access all the data. We used MS Forms as it provided a suitable integrated web interface and data collection tool for the survey. The data within MS Forms ‘are encrypted both at rest and in transit’ and are stored on a European Server, compliant with UK General Data Protection Regulation (UK GPDR).^26,27^

When the active period for the survey ended, the response summary information and the individual responses of the complete surveys were exported from MS Forms directly to A.R.’s DataStore allocation, a secured and password-protected area at the University of Edinburgh, in accordance with their data handling policies.^28–30^

Individual responses were kept until February 2023, then destroyed in accordance with the University of Edinburgh policy for destroying archived research data.^31,32^

Closed questions were analysed using descriptive statistics (counts and percentages) in SAS 9.4.^33^ All data were analysed by A.R.

Thematic analysis^34,35^ was used to generate themes from the open-ended questions using NVivo^®^ January 2022 (Release 1.6.1).^36^ Participants had the freedom to write as much as they wanted and express several opinions for any given topic. The free-text data were initially coded solely by A.R. These themes were then reviewed, refined and finalised on 7 March 2023, through discussion with the multi-disciplinary research team, to ensure valuable perspectives were included and to help reduce the subjectivity of the findings (S.C.L., C.J.W. and T.J.).

The results of this study helped us to understand the views of UK researchers regarding their experiences with the creation and release of de-identified/anonymised clinical trial datasets, the generation and use of re-identification risk scores, and their views about wider aspects of re-identification risks.

## Results

The pilot survey was active from 6 June 2022 to 29 August 2022 inclusive, and the main survey was active from 13 September 2022 to 19 October 2022. There were no changes made to the survey between the pilot and main phase. We obtained 52 consented participants in total of which 38 were eligible because they identified as being based in the United Kingdom. No data from the eligible participants were excluded from the analysis. Notably, 32 (84%) participants were associated with a UKCRC-registered CTU. The average time to complete the survey was 18 min and all participants reached the end of the survey, so there is no missing data to report. The most common role was statistician (including senior statistician; 17 (44%)), followed by Director/Senior Manager (6 (16%)) and principal investigator (4 (11%)). However, 27 (71%) participants had at least 6 years of experience in their employed role at the moment they took the survey. Table 1 has more details on the participant characteristics.

**Table 1.** Participant characteristics and experience and documentation used on the creation/release of de-identified/anonymised datasets.

Parameters	Participants N = 38 n (%)
Place of work	
UKCRC-registered CTU	32 (84)
Other^a^	6 (16)
Employed role	
Director/Senior Manager	6 (16)
Principal Investigator	4 (11)
Researcher (Research fellow/assistant)	2 (5)
Senior Researcher	1 (3)
Statistician	7 (18)
Senior Statistician	10 (26)
Trial Manager/Coordinator	1 (3)
Senior Trial Manager/Coordinator	1 (3)
Other	6 (16)
Years of experience in employed role	
0–2	3 (8)
3–5	8 (21)
6–10	9 (24)
>10	18 (47)
Involvement with the de-identification/anonymisation tasks^b^	
○ Creation/generation of de-identified/anonymised dataset	27 (71)
○ Evaluation/assessment/peer review of de-identified/anonymised dataset	15 (40)
○ Approval of the release of de-identified/anonymised dataset	21 (55)
○ Generation/evaluation/assessment of de-identified/anonymised dataset re-identification risk	10 (26)
○ Uploading/maintenance/distribution of de-identified/anonymised dataset	14 (37)
○ Other^c^	2 (5)
Years of experience in de-identification/anonymisation	
0–2	14 (37)
3–5	12 (32)
6–10	6 (16)
>10	6 (16)
Documents used	
Only internally developed documents/guidance	8 (21)
Only externally sourced documents/guidance	4 (11)
Both internal and external documents/guidance	21 (55)
Other^d^	5 (13)
Topics covered by the internally developed documents/guidance^e^	
•The process of how to de-identify/anonymise clinical trial datasets	
Yes (documents/guidance implemented)	16 (55)
Yes (but document/guidance under construction)	8 (28)
No (this process is not covered)	4 (14)
No response	1 (3)
•The process for releasing de-identified/anonymised clinical trial datasets	
Yes (documents/guidance implemented)	18 (62)
Yes (but document/guidance under construction)	6 (21)
No (this process is not covered)	3 (10)
No response	2 (7)
•The assessment of the re-identification risk of the de-identified/anonymised clinical trial datasets	
Yes (documents/guidance implemented)	4 (14)
Yes (but document/guidance under construction)	7 (24)
No (this process is not covered)	15 (52)
No response	3 (10)
Process use for releasing de-identified/anonymised clinical trial data	
Only de-identification, under controlled access	13 (34)
De-identification followed by anonymisation, under controlled access	18 (47)
Only de-identification, under open access	1 (3)
De-identification followed by anonymisation, under open access	1 (3)
Other^f^	5 (13)

a Other = 1 CRO, 1 Medical School, 1 Retired, 2 University, 1 UoE

b Participants were allowed to mark multiple types of involvement.

c Other = not directly involved

d Other = 3 none/not applicable, 1 ‘NHSD & CAG advise relating to what is classed as identifiable and sensitive’ and 1 ‘Internal and also review the funding body guidance for whoever funded the study’

e Only applicable to the 29 participants who answered in the previous question ‘Only internally developed documents/guidance’ and ‘Both internal and external documents/guidance’

f Other = 2 not applicable, 1 ‘De-identification followed by some aspects of anonymisation, under controlled access (pseudonymisation: there is some manipulation, but the data could not be described as anonymised, and the link may not be destroyed)’, 1 ‘Defining these processes is a work in progress for us, but any data releases would be under controlled access’, 1 ‘varies depending on the risk’

The most common involvement with the de-identification/anonymisation datasets was with their creation/generation (27 participants (71%)) and approval (21 participants (55%)) of the release of de-identified/anonymised datasets. Notably, 27 (71%) participants were involved in more than one task. However, 24 (63%) participants had at least 3 years of experience in dealing with de-identification/anonymisation datasets. In addition, 35 (92%) participants used documentation/guidance for de-identification/anonymisation, of which 21 (60% of 35) participants used both internal and external documents/guidance. Moreover, 24 out of 29 (83%) of the internally generated documentation (either implemented or under construction) covered the topic of how to de-identify/anonymise datasets, also 24 out of 29 (83%) covered the releasing of de-identified/anonymised datasets and 11 out of 29 (38%) covered the assessment of the re-identification risk. De-identification, followed by anonymisation and then fulfilling data holders’ requirements before access was granted (controlled access), was the most common process for releasing the datasets with 18 responses (47%). Further detail is presented in Table 1.

Views on the process of de-identifying/anonymising datasets were asked. From thematic analysis of data from 38 participants, we obtained 81 separate opinions on de-identifying/anonymising datasets. However, 63 expressed a negative sentiment and we categorised them as follows: long process (13), lack of advice (13), data constraints and keeping utility (10), lack of resources (9), risky process (8), difficult process (4), non-reversible process (2), not applicable to old datasets (2), forced process (1), and data requestors not willing to pay/wait (1). Moreover, 17 opinions were of a positive nature and were categorised as: straightforward process (9), others do it (3), non-risky process (2), sharing with trusted bona fide researchers (2) and guidance available (1). Table 2 has representative quotes for each category.

**Table 2.** Opinions about the process of de-identifying/anonymising datasets.

Name of code	No. of comments	Description/representative quote
Opinion process of de-identifying/anonymising	81^a^	In your opinion, how was your experience in the process of de-identifying/anonymising clinical trials datasets? (e.g. what have worked? what have not worked? any concerns? any assurances?)
+ Is data used for secondary research?	1	Is there any value of doing this?
+ Negative	63^b^	
Data constraints and keeping utility	10	Age and gender are often key parts of an analysis, which we can’t remove / do the de-identification without rendering the database meaningless
Difficult process	4	This can be a challenging process
Forced process	1	Release of anonymised data was required for legal reasons
Lack of advice	13	It was difficult to get specific advice on how to de-identify and anonymise data. Especially on assessment of anonymised data
Lack of resources	9	we do not have the resources for this–it is hard to fund as it occurs after the end of a study grant. / You need to find someone who can understand the original data collection and databases and who has the time and skill to identify which fields need to be removed
Data requestors not willing to pay/wait	1	It doesn’t happen often, and mainly, people don’t want the data badly enough to pay for the work or wait.
Not applicable to old datasets	2	We work with older datasets so the technical side of de-identifying data that was collected at a point when none of the current legislation was in place is demanding
Long process	13	Generally time consuming. / It takes a long time to do it properly
Non-reversible process	2	I recall MRC guidance is to generate a new patient reference number and delete code to generate that number. In practice this is impractical as if the requestor needs clarification on an element of the data being shared you have lost the ability to identify that patient within your own ‘parent’ dataset
Risky process	8	This makes deidentifying/ anonymising an unnerving experience
+ Positive	17^b^	
Non-risky process	2	however in my opinion it is almost never that this puts participants at risk
Sharing with trusted bona fide researchers	2	We share them with bona fide researchers, with good research ideas, and this has not caused us concern
Others do it	3	Others have performed the process
Straight forward process	9	It was fairly straightforward
Guidance available	1	use the ICO guidelines

a Only main nodes added (+)

b Only children nodes added

Also, opinions regarding the process of releasing datasets were sought. In total, 38 participants provided 59 opinions. However, 24 expressed negative sentiments, such as issues with data sharing agreements (7), lack of resources (5), risky task (5), complex requests (2), paperwork/red tape/regulation (2) and no demand (2). Meanwhile, 29 opinions reflected positive sentiments, such as easy process (7), easy data transfer (6), committees/expert approvals (5), use of data repositories (4), use of controlled environment (3), vetoing researchers (3) and open access (1). Table 3 has further details.

**Table 3.** Opinions about the process of release datasets.

Name of code	No. of comments	Description/representative quote
Opinion process of release datasets	59^a^	In your opinion, how was your experience in the process of releasing de-identified/anonymised clinical trials datasets? (e.g. what have worked? what have not worked? any concerns? any assurances?)
+ Done by others	3	Having gone through the de-identifying phase the release was overseen by others
+ Not done this yet	3	Have not done this yet
+ Negative	24^b^	
Complex requests	2	there is considerable conversation regarding the manipulation to end up at the dataset
Process improving	1	The process here is getting better
Issues with data sharing agreements	7	Main issue has been regulatory/contracts rather than data process
Paperwork/red tape/regulation	2	More form filling in / Too much regulation
Lack of resources	5	I have found this process fairly labour intensive and resource for these tasks is generally not costed
No demand	2	no other groups have requested trial datasets to date
Risky task	5	it is a source of worry that we might inadvertently do something illegal
+ Positive	29^b^	
Committees/expert approvals	5	this process simply requires a robust documented process for deciding to release the data
Use of controlled environment	3	data should be released via a secure research environment
Use of data repositories	4	Use of institutional repository
Easy data transfer	6	Once an appropriate transfer method was identified and agreed, releasing the data set was straightforward
Easy process	7	Very easy. But if you trial is not controversial or current
Open access	1	This is not a concern if you are releasing open-access
Vetoing researchers	3	I feel comfortable releasing to bona fide researchers with good research ideas

a Only main nodes added (+)

b Only children nodes added

Opinions on the experience of maintaining released de-identified/anonymised datasets were collected: 12 participants expressed that they did not have experience or that it was not a necessary process. From the 26 participants who had experience in this area, we collected 11 negative opinions of this process being a burden (no resources (3), dynamic process (3), issue with contracts (2), bespoke process (1), difficult to keep links (1) and difficult to keep process for access (1)). The only positive opinion was that the maintenance was done for free by the participant’s institution (details in Table 4).

**Table 4.** Opinions about the process of maintaining datasets.

Name of code	No. of comments	Description/representative quote
Experience of maintaining	24^a^	In your opinion, how was your experience in the process of maintaining released de-identified/anonymised clinical trials datasets? (e.g. what have worked? what have not worked? any concerns? any assurances?)
+ No experience	9	No experience of this
+ No necessary	3	We don’t maintain released datasets
+ Negative/burden	11^b^	
Bespoke process	1	there was no standardised way of maintaining de-identified datasets… anyone tasked with preparing a data release would first have to assess previous work. This was a large waste of time
Issue with contracts	2	Main issue has been regulatory/contracts rather than data process
Dynamic process	3	Using a controlled process means that we have much more control over the data regarding version control and ensuring that the data remains accessible using current technology
Difficult to keep links	1	Need to have correct processes in place to ensure that additional data can be linked with the existing data released without compromising data integrity or de-identification
Difficult to keep process for access	1	Some trial datasets that are still potentially available from studies I have worked on are password protected and the staff who knew the passwords have left without remaining staff having requested these.
No resources	3	It is difficult to arrange a process for maintaining controlled access that is available long-term and affordable
+ Positive/constructive	1^b^	
Keep for free by organisation	1	Kept in university

a Only main nodes added (+)

b Only children nodes added

In addition, 38 (100%) of the participants did not use any kind of re-identification risk score, this was distributed as follows: 18 (47%) participants had never heard of re-identification risk scores while 9 (24%) and 11 (29%) of the participants have, respectively, ‘heard about them, but were not so sure what they are’ and ‘have a general understanding, but did not use them’. This last group expressed a lack of relevant training ((11/11) 100%), a lack of time ((8/11) 73%) and a lack of funding ((4/11), 45%) as the main barriers for not using re-identification risk scores (see Table 5). Further questions exploring the views about re-identification risks scores were not answered by any participant because they were only applicable if the participants answered that they had a ‘good or strong’ understanding of re-identification risk scores and used them ‘sometimes or frequently’.

**Table 5.** Views about re-identification risks scores.

	Participants N = 38 n (%)
Awareness of re-identification risk scores for assisting in the release of de-identified/anonymised clinical trial datasets	
I have never heard of them	18 (47)
I have heard about them, but I am not so sure what they are	9 (24)
I have a general understanding but do not use them	11 (29)
I have a good understanding and use them sometimes	0
I have a strong understanding and use them frequently	0
What are the barrier for not using the re-identification risk scores^a^	
Lack of funding	5 (45)
Lack of relevant training	11 (100)
Lack of time	8 (73)
Other^b^	4 (36)

a Only applicable to the 11 participants who answered in the previous question ‘I have a general understanding, but do not use them’. Participants were allowed to mark multiple barriers.

b Other =1 ‘I’m not convinced they are necessary’, 1 ‘Possible lack of benefit if following existing good process’, 1 ‘Specific examples based around clinical trial data’, 1 ‘study context, harder to conceptualise this metric than something descriptive. I’m fully aware of the irony that a statistician is essentially saying use free text here! I think they do have something to offer but need context’.

Note: As No one selected ‘I have a good understanding, and use them sometimes’ or ‘I have a strong understanding, and use them frequently’ in Q15, Q16–Q20 were no longer feasible and the survey automatically skipped to Q21 for the participant who answered ‘I have a general understanding, but do not use them’, and to Q22 for the participants who answered ‘I have never heard of them’ or ‘I have heard about them, but I am not so sure what they are’

Regarding the wider aspects of re-identification risk, we attempted to identify which concerns related to the de-identified/anonymised datasets’ properties were known to the researchers before release: 98% of the researchers always or often considered the data format, 71% always or often thought about the data uniqueness and 95% always or often contemplated the sensitivity of the data (Supplementary Figure 1). We also asked about the concerns around the release environment for de-identified/anonymised datasets and 52% of researchers always or often considered motivations to launch a re-identification attack on the datasets, 36% always or often considered the existence of auxiliary information to enable a re-identification attack, 26% always or often thought about the geographical location of the release, 61% contemplated consequences to individuals and 61% considered consequences to organisations if a successful re-identification attack occurred (Supplementary Figure 2).

Finally, we asked about any other aspects researchers considered before the release of anonymised/de-identified datasets. In total, 10 participants expressed concerns around the following themes: Any benefits of data release (3), contracts concerns (2), avoidance of open access (1), ethical approval concerns (1), expertise of data releaser (1), patients’ expectation (1) and reasons to release data (1), and 30 participants mentioned steps/checks before data release which included verification of a good research question (8), vetoing researchers (7), consideration of the probability of re-identification (5), presence of any data uniqueness (4), execution of final checks (3) and provision of storage guidance for the released data (3) (Table 6).

**Table 6.** Any other aspects you consider before release.

Name of code	N	Description/representative quote
Any other aspects you consider before release	40^a^	Any other aspects you consider before you release de-identified /anonymised clinical trials data.
+ Concerns	10^b^	
Avoidance of open access	1	We’ve avoided true open access data release
Any benefits of data release	3	What are the potential scientific and societal benefits from release?
Contract concerns	2	Is the contract appropriate and does it make clear the duties of the requestor?
Ethical approval concerns	1	Ethics worries me
Expertise of data releaser	1	release is down to the highest data owner which, in our case, is the main trial statistician (with oversight). I wouldn’t leave this decision to techies
Patients’ expectation	1	Patient expectations of how their data would be handled (was consent obtained, how was the consent worded, what do patient representatives suggest about sharing that data specifically?)
Reasons to release data	1	(is it to be included in further meta-analysis, for potential secondary analysis, or are just released for ‘accountability’/because journals asked us to). What was said in the consent form about data use/sharing?
+ Steps/checks	30^b^	
Presence of any data uniqueness	4	Any release of special category data especially if there is a uniqueness inherent in the data
Execution of final checks	3	Robust checking of dataset prior to release to try to mitigate any of the risks
Verification of a good research question	8	favouring controlled release to groups that have a plan of what to do with the data
Provision of storage guidance for the released data	3	We give guidance on how the data should be stored when they are received, to reduce the risk of them being held insecurely
Consideration of the probability of re-identification	5	There is always a fear that you feel that the data, even with rigorous anonymous checks, by several people, that someone will be able to be identified with the data
Vetoing researchers	7	We ask for details of who the researchers are, and what their question is. We only release to bona fide researchers, with good research questions

a Only main nodes added (+)

b Only children nodes added

## Discussion

Researchers participating in this study belong to a group experienced in the conduct of clinical trials and in the processes associated with the preparation and release of de-identified/anonymised clinical trial datasets.

The dominant activities that respondents engaged in were the creation of the de-identified/anonymised datasets followed by the approval for release, while maintenance and peer review of the de-identified/anonymised datasets do not seem to be as common. This could be explained by the participant’s profile, as statisticians and trial managers are more likely to take part in the creation of de-identified/anonymised datasets,^37,38^ while IT specialists tend to be involved in the uploading and maintenance of the datasets, and this group is not represented in our results (despite the survey being open to it). Another possible explanation is that we assumed sharable datasets would be created at the end of a study, ready to be sent out on request.^39–41^ However, in reality, sharable datasets are often created on demand, eliminating the need for maintenance.^42^

It is encouraging that 92% of the participants used some sort of documentation (either external or internal) for the de-identification/anonymisation process, with the process of how to de-identify/anonymise and release datasets being highly represented; conversely, the implementation of risk evaluation is only modestly represented in these documents.

The most common overall process for the release of de-identified/anonymised clinical trial datasets, as reported by participants, was ‘de-identification followed by anonymisation under controlled access’, here clinical trial datasets are de-identified (key items stripped from the dataset), this is followed by data manipulation techniques to further anonymise the datasets and finally, datasets are released via the implementation of, for example, data sharing agreements, the location of the datasets behind secure access barriers or the identification and vetoing of secondary researchers and their research ideas. This process matches what we found in our previously published systematic scoping review.^43^ This is promising because researchers are following the proposed recommendations/guidelines, which over time are providing a robust process, as evidenced by the fact that we do not yet have any known cases of a successful re-identification attack in the United Kingdom in clinical trial datasets.

When we explored researchers’ opinions on the process of de-identifying/anonymising and maintaining datasets, negative sentiments seemed to dominate. Opinions on the data release process were balanced between positive and negative views. This suggests that de-identifying/anonymising the data is more troublesome than releasing it. This could be explained by the time in which data preparation and sharing activities are occurring. These activities tend to happen at the end of the studies, when the budget has been expended and the teams have pressures from other live projects.^37^ The International Committee of Medical Journal Editors (ICMJE) have acknowledged this situation and they are recommending any trials that started enrolling participants after the 1 January 2019 must have a data sharing plan in the trial’s registration.^41^ However, at present, it might be premature to expect to see the emerging impact of that recommendation. Time is not the only constraint; it is known that de-identification and anonymisation of datasets are potentially complex and getting it wrong could have profound consequences.^44,45^

Regarding re-identification risk scores, it was expected that researchers would not know about how to calculate them, due to two main reasons: first, they are briefly described in the current guidance documents for clinical trials,^43^ and second, the proposed re-identification risk scores come from health records management, so they are not common knowledge among clinical trialists. The small group of participants, who were aware of re-identification risk scores but were not using them, cited the primary reasons as a lack of training and time. Addressing the lack of training is an aspect that could be considered. This is an emerging topic within clinical trials where there are research and training gaps because researchers need a clear and tailored tool that they can use to estimate the re-identification risk for datasets.

We attempted to explore concerns about known parameters that could affect the re-identification risk,^46–50^ using variables related to the datasets and to their release environments. We observed that even with no formal training, some researchers are already intuitively addressing these parameters and thinking of ways of mitigating their impact during the preparation and before the release of de-identified/anonymised datasets. Of course, robust guidance and availability of training could help to increase the level of engagement with the features that could affect re-identification risk.

Finally, we invited comment on issues that were not addressed in the rest of the survey, but notably the responses did not identify any new practices or concerns.

### Comparison with existing literature

The report by Humphreys et al.^10^ dealt with issues regarding wider aspects of data sharing for clinical trials and it commented that better guidance, more resources and training are required to fulfil data sharing from clinical trials. Our survey results agree with these findings. So, this is not an exclusive issue for de-identified/anonymised datasets.

Naudet et al.^51^ reported a low incidence of sharing of de-identified/anonymised clinical trial datasets. They explained that the main reasons for the ICMJE data sharing policy not being implemented are lack of resources and training, lack of unified concepts (e.g. multiple definitions for anonymisation), real or perceived risk and the need to protect the interests of researchers and patients. This suggests that the barriers are not only researchers’ opinions but also a reality. Currently, promises to eventually share data are not being kept.^42,52^ Therefore, a future where data sharing is the norm is still out of reach until these issues are mitigated.

Humphreys et al.^10^ highlighted re-identification risk as a key issue, but they did not describe how this risk should be calculated or quantify an acceptable level of risk. Our study is a first step to encourage a research stream for the underdeveloped area of re-identification risk estimation on clinical trial datasets.

### Strengths and limitations

We sent the survey to mailing lists involving large numbers of people to maximise the chance of responses from individuals eligible for the survey. However, many of the people in these mailing lists would not have been eligible. We therefore could not investigate the response rate or the response bias of the survey. We collected responses from 38 participants, which, according to the methodology used in this research, is sufficient to reach data saturation with respect to opinions, as we exceeded the minimum of between 12 and 30 survey responses. However, we may not have heard all opinions because, for example, we did not receive surveys from every UKCRC-registered CTU (population size n = 52). In addition, the CTUs represented in our sample may be a biased subset. We also do not know if the participants filling out the survey were speaking solely about their personal experience or if they were representing their CTU. However, the researchers who participated in this study were experienced and appeared to have relevant hands-on experience of the process of de-identification/anonymisation of clinical trial datasets. This gives strength to the results. However, eligible individuals self-identified as experienced in the subject matter and, we did not assess the details of this experience; instead, we inquired about the number of years of experience in de-identification/anonymisation.

All eligible individuals reached the end of the survey, and this is evidence of highly motivated participants interested in the topic of the survey. Such highly motivated individuals could potentially provide mostly positive experiences, and we might not have fully engaged with researchers who have done de-identification/anonymisation and have had adverse experiences or difficulties in this area. Nevertheless, we recorded a high incidence of negative sentiments.

As our resources were limited, we based the survey in the United Kingdom due to the complexities of ethical approvals. We could not predict where our potential international participants were going to be based and this restricted the ethical approval application to only the United Kingdom. Therefore, it was not possible to explore what is happening in other countries. To our knowledge, this has not been studied in other settings and future research addressing this gap would be valuable to confirm the generalisability of these findings. In this regard, we are sharing our full protocol and survey. Of course, many of the issues highlighted in this study are common global problems, so this UK study could be relevant to the wider research community.

The themes were manually coded and, therefore subjective; however, other reviewers sense-checked the coding and any disagreements were resolved via discussion within the research team.

This study is covering an emerging part of clinical trials research, for which even consensus about the definition of anonymisation does not exist.^43^ To avoid misinterpretations in the survey, we provided definitions in the body of the survey to counter this issue. However, it cannot be ruled out that some of reported practices could be related to the sharing of pseudonymised data, as indicated by the use of controlled access.

## Conclusion

It is positive to see that the majority of responders reported using documented processes for de-identification and anonymisation of clinical trial datasets. However, our survey results clearly indicate that there are still gaps in the areas of guidance, resources and training to fulfil sharing requests of de-identified/anonymised clinical trial datasets. In addition, the investigation of applications of re-identification risk scores on de-identified/anonymised clinical trial datasets could help with the development of an objective process to assess the re-identification risks and probabilities of re-identification attacks, which in turn can harmonise efforts towards more secure de-identified/anonymised datasets.

Meanwhile funders and sponsors should continue to foster and support activities regarding the preparation of de-identified/anonymised clinical trial datasets with the intention to share, such as training and funded time for these tasks.

## Supplemental Material

sj-docx-1-ctj-10.1177_17407745241259086 – Supplemental material for A survey on UK researchers’ views regarding their experiences with the de-identification, anonymisation, release methods and re-identification risk estimation for clinical trial datasets Supplemental material, sj-docx-1-ctj-10.1177_17407745241259086 for A survey on UK researchers’ views regarding their experiences with the de-identification, anonymisation, release methods and re-identification risk estimation for clinical trial datasets by Aryelly Rodriguez, Steff C Lewis, Sandra Eldridge, Tracy Jackson and Christopher J Weir in Clinical Trials

sj-docx-4-ctj-10.1177_17407745241259086 – Supplemental material for A survey on UK researchers’ views regarding their experiences with the de-identification, anonymisation, release methods and re-identification risk estimation for clinical trial datasets Supplemental material, sj-docx-4-ctj-10.1177_17407745241259086 for A survey on UK researchers’ views regarding their experiences with the de-identification, anonymisation, release methods and re-identification risk estimation for clinical trial datasets by Aryelly Rodriguez, Steff C Lewis, Sandra Eldridge, Tracy Jackson and Christopher J Weir in Clinical Trials

sj-docx-5-ctj-10.1177_17407745241259086 – Supplemental material for A survey on UK researchers’ views regarding their experiences with the de-identification, anonymisation, release methods and re-identification risk estimation for clinical trial datasets Supplemental material, sj-docx-5-ctj-10.1177_17407745241259086 for A survey on UK researchers’ views regarding their experiences with the de-identification, anonymisation, release methods and re-identification risk estimation for clinical trial datasets by Aryelly Rodriguez, Steff C Lewis, Sandra Eldridge, Tracy Jackson and Christopher J Weir in Clinical Trials

sj-docx-6-ctj-10.1177_17407745241259086 – Supplemental material for A survey on UK researchers’ views regarding their experiences with the de-identification, anonymisation, release methods and re-identification risk estimation for clinical trial datasets Supplemental material, sj-docx-6-ctj-10.1177_17407745241259086 for A survey on UK researchers’ views regarding their experiences with the de-identification, anonymisation, release methods and re-identification risk estimation for clinical trial datasets by Aryelly Rodriguez, Steff C Lewis, Sandra Eldridge, Tracy Jackson and Christopher J Weir in Clinical Trials

sj-pdf-2-ctj-10.1177_17407745241259086 – Supplemental material for A survey on UK researchers’ views regarding their experiences with the de-identification, anonymisation, release methods and re-identification risk estimation for clinical trial datasets Supplemental material, sj-pdf-2-ctj-10.1177_17407745241259086 for A survey on UK researchers’ views regarding their experiences with the de-identification, anonymisation, release methods and re-identification risk estimation for clinical trial datasets by Aryelly Rodriguez, Steff C Lewis, Sandra Eldridge, Tracy Jackson and Christopher J Weir in Clinical Trials

sj-pdf-3-ctj-10.1177_17407745241259086 – Supplemental material for A survey on UK researchers’ views regarding their experiences with the de-identification, anonymisation, release methods and re-identification risk estimation for clinical trial datasets Supplemental material, sj-pdf-3-ctj-10.1177_17407745241259086 for A survey on UK researchers’ views regarding their experiences with the de-identification, anonymisation, release methods and re-identification risk estimation for clinical trial datasets by Aryelly Rodriguez, Steff C Lewis, Sandra Eldridge, Tracy Jackson and Christopher J Weir in Clinical Trials

sj-pdf-7-ctj-10.1177_17407745241259086 – Supplemental material for A survey on UK researchers’ views regarding their experiences with the de-identification, anonymisation, release methods and re-identification risk estimation for clinical trial datasets Supplemental material, sj-pdf-7-ctj-10.1177_17407745241259086 for A survey on UK researchers’ views regarding their experiences with the de-identification, anonymisation, release methods and re-identification risk estimation for clinical trial datasets by Aryelly Rodriguez, Steff C Lewis, Sandra Eldridge, Tracy Jackson and Christopher J Weir in Clinical Trials

sj-pdf-8-ctj-10.1177_17407745241259086 – Supplemental material for A survey on UK researchers’ views regarding their experiences with the de-identification, anonymisation, release methods and re-identification risk estimation for clinical trial datasets Supplemental material, sj-pdf-8-ctj-10.1177_17407745241259086 for A survey on UK researchers’ views regarding their experiences with the de-identification, anonymisation, release methods and re-identification risk estimation for clinical trial datasets by Aryelly Rodriguez, Steff C Lewis, Sandra Eldridge, Tracy Jackson and Christopher J Weir in Clinical Trials

sj-xlsx-9-ctj-10.1177_17407745241259086 – Supplemental material for A survey on UK researchers’ views regarding their experiences with the de-identification, anonymisation, release methods and re-identification risk estimation for clinical trial datasets Supplemental material, sj-xlsx-9-ctj-10.1177_17407745241259086 for A survey on UK researchers’ views regarding their experiences with the de-identification, anonymisation, release methods and re-identification risk estimation for clinical trial datasets by Aryelly Rodriguez, Steff C Lewis, Sandra Eldridge, Tracy Jackson and Christopher J Weir in Clinical Trials
