# Supplementary material for: A survey on UK researchers’ views regarding their experiences with the de-identification, anonymisation, release methods and re-identification risk estimation for clinical trial datasets
Source: Clin Trials. 2024 Jun 19;22(1):11–23. doi: 10.1177/17407745241259086 (PMC11809122; doi:10.1177/17407745241259086)
Supplement: sj-docx-4-ctj-10.1177_17407745241259086 – Supplemental material for A survey on UK researchers’ views regarding their experiences with the de-identification, anonymisation, release methods and re-identification risk estimation for clinical trial datasets [file sj-docx-4-ctj-10.1177_17407745241259086.docx]

***Additional file 3 A checklist of Questions for Designing a Survey Study Plan***

***(Extracted from Chapter 8 in Research Design by John Creswell and J. David Creswell, 5^th^ edition.)***

|  |  |  |
| --- | --- | --- |
| Item ID | Item description | Protocol compliance |
| 1 | Is the purpose of the survey stated? | Yes |
| 2 | Are the reasons for choosing the design mentioned? | Yes |
| 3 | Is the nature of the survey (cross-sectional vs. longitudinal) identified | Yes |
| 4 | Is the population and its size mentioned? | Yes |
| 5 | Will the population be stratified? If so how? | Yes |
| 6 | How many people will be in the sample? On what basis was this size chosen? | Yes |
| 7 | What will be the procedure for sampling these individuals (e.g. random, non-random)? | Yes |
| 8 | What instrument will be used in the survey? Who developed the instrument? | Not applicable |
| 9 | What are the content areas addressed in the survey? The Scales | Yes |
| 10 | What procedure will be used to pilot or field test the survey? | Yes |
| 11 | What is the timeline for administering the survey? | Yes |
| 12 | What are the variables in the survey? | Yes |
| 13 | How do these variables cross-reference with the research questions and items on the survey? | Yes |
| 14 | What specific steps will be taken in data analysis to do the following |  |
| 14a | Analyse returns? | Yes |
| 14b | Check for response bias? | Yes |
| 14c | Conduct a descriptive analysis? | Yes |
| 14d | Collapse items into scales? | Yes |
| 14e | Check for reliability of scales? | Not applicable |
| 14f | Run inferential statistics to answer research questions or assess practical implications of the results? | Not applicable |
| 15 | How will the results be interpreted? | Yes |
