# Supplementary figures and images for: A survey on UK researchers’ views regarding their experiences with the de-identification, anonymisation, release methods and re-identification risk estimation for clinical trial datasets
Source: Clin Trials. 2024 Jun 19;22(1):11–23. doi: 10.1177/17407745241259086 (PMC11809122; doi:10.1177/17407745241259086)

Additional file 4 Survey data collection process.

| **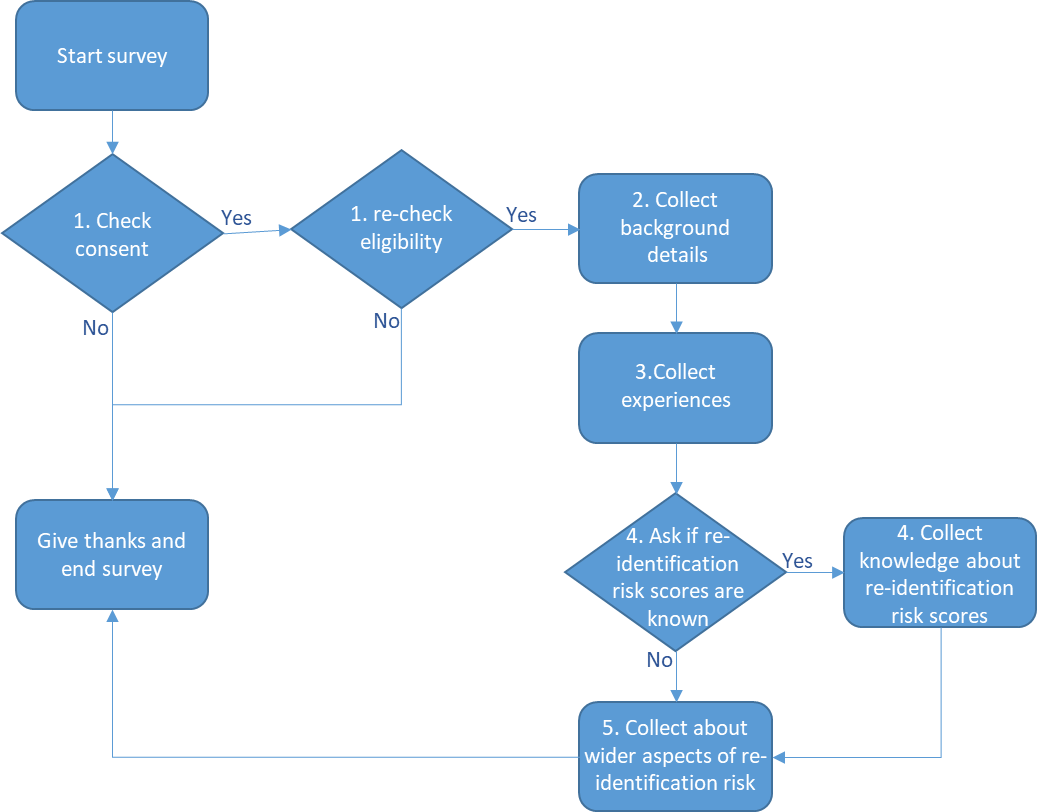** |
| --- |
| **Survey data collection process.** |

Supplement: sj-docx-5-ctj-10.1177_17407745241259086 – Supplemental material for A survey on UK researchers’ views regarding their experiences with the de-identification, anonymisation, release methods and re-identification risk estimation for clinical trial datasets [file sj-docx-5-ctj-10.1177_17407745241259086.docx]
