# Supplementary material for: A survey on UK researchers’ views regarding their experiences with the de-identification, anonymisation, release methods and re-identification risk estimation for clinical trial datasets
Source: Clin Trials. 2024 Jun 19;22(1):11–23. doi: 10.1177/17407745241259086 (PMC11809122; doi:10.1177/17407745241259086)
Supplement: sj-docx-6-ctj-10.1177_17407745241259086 – Supplemental material for A survey on UK researchers’ views regarding their experiences with the de-identification, anonymisation, release methods and re-identification risk estimation for clinical trial datasets [file sj-docx-6-ctj-10.1177_17407745241259086.docx]

Additional file 5 Invitation (email or print-out) for survey

**An online survey of UK researchers' views on sharing anonymised clinical trials datasets. - Survey closing on the 19th October 2022**

There are increasing pressures for anonymised datasets from clinical trials to be shared across the scientific community. However there is no a single standardised set of recommendations on how to anonymise and prepare clinical trial datasets for sharing and an ever increasing number of anonymised clinical trials datasets are becoming available for secondary research.

As part of my PhD research, I'm working with my supervisors the University of Edinburgh to examine this topic and would like to hear the views and experiences of researchers in the UK about de-identification, anonymisation, release methods and re-identification risk estimation for clinical trials datasets.

It would be very useful to hear your views. The survey is available until the 19th October 2022.
Please use the link or the QR code for more details about this survey and thank you!

<https://forms.office.com/r/0aTLCVqmE3>


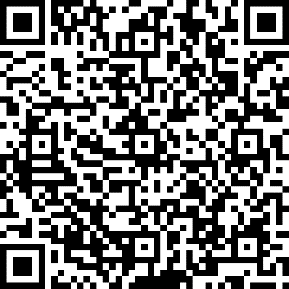


The survey will take about 15 minutes to complete and all responses will be anonymised and kept confidential.

Should you have any further questions, please contact me at:

Aryelly Rodriguez

Statistician/PhD candidate, Edinburgh Clinical Trials Unit, The University of Edinburgh, UK.

aryelly.rodriguez@ed.ac.uk
