# Supplementary material for: A survey on UK researchers’ views regarding their experiences with the de-identification, anonymisation, release methods and re-identification risk estimation for clinical trial datasets
Source: Clin Trials. 2024 Jun 19;22(1):11–23. doi: 10.1177/17407745241259086 (PMC11809122; doi:10.1177/17407745241259086)
Supplement: sj-pdf-2-ctj-10.1177_17407745241259086 – Supplemental material for A survey on UK researchers’ views regarding their experiences with the de-identification, anonymisation, release methods and re-identification risk estimation for clinical trial datasets [file sj-pdf-2-ctj-10.1177_17407745241259086.pdf]

What are the UK researchers' views regarding their experiences with the de-identification, anonymisation, release methods and re-identification risk estimation for clinical trials datasets? A study protocol

Rodriguez A[1], Lewis SC[1], Eldridge S[2], Jackson T[3], Weir CJ[1]

[1]Edinburgh Clinical Trials Unit, Usher Institute, the University of Edinburgh

[2]Pragmatic Clinical Trials Unit, Blizard Institute, Barts and the London School of Medicine and Dentistry, Queen Mary University of London

[3]Asthma UK Centre for Applied Research, Usher Institute, the University of Edinburgh

Correspondence:

Ms Aryelly Rodriguez

Edinburgh Clinical Trials Unit, the University of Edinburgh

Level 2, Nine Edinburgh BioQuarter,

9 Little France Road, Edinburgh, EH16 4UX

Emails: aryelly.rodriguez@ed.ac.uk, steff.lewis@ed.ac.uk, christopher.weir@ed.ac.uk, Tracy.Jackson@ed.ac.uk, s.eldridge@qmul.ac.uk

What are the UK researchers' views regarding their experiences with the de-identification, anonymisation, release methods and re-identification risk estimation for clinical trials datasets? A study protocol

Abstract

There are increasing pressures for anonymised datasets from clinical trials to be shared across the scientific community. However there is no a single standardised set of recommendations on how to anonymise and prepare clinical trial datasets for sharing and an ever increasing number of anonymised clinical trials datasets are becoming available for secondary research. Therefore, this study aims to explore the current views and experiences of researchers in the UK about de-identification, anonymisation, release methods and re-identification risk estimation for clinical trials datasets.

Key Words: Clinical Trials | Data Anonymisation | Re-identification | De-identification | Datasets | Re-identification risk

Definitions

**Anonymisation**	A data set would be considered anonymised if it has been de-identified and then subsequent data manipulation/steps have been taken to further protect the dataset, for example, if a privacy model has been applied (e.g. k-anonymity) or the link with the original non anonymised dataset has been destroyed and this action cannot be reversed.
**De-identification**	Removal of all personal health information and all other indirect identifiers which could lead to the identification of an individual. The most common de-identification methods are:<br>1. HIPPA (US Health Insurance Portability and Accountability Act of 1996) Safe harbour, in which 18 identifiers are removed from the datasets [1] [2]<br>2. Hrynaszkiewicz et al. [3] proposed an enhanced removal of potential identifiers which are commonly present in clinical trials datasets.
**Controlled Access**	Datasets that can only be accessed if permission is granted by the data holders via their internal procedures.
**Open Access:**	Datasets that can be accessed without any or minimal restrictions imposed by the data holders.
**Publicly available datasets**	Data sets that are discoverable and available for sharing via open or controlled access, this data can be located on central repositories or with individual institutions/researchers
**Re-identification risk score**	Estimated probability of any given individual being re-identified from an anonymised/de-identified dataset. The re-identification risk score depends on the variables available in the dataset, the number of observations in the dataset and on the strategy used to attack the dataset (prosecutor or journalist scenario).
**Prosecutor scenario**	If the adversary knows that a target individual (for whom identifiers are known) is in the publicly available dataset (released anonymised and/or de-identified) we are under prosecutor re-identification risk scores. This scenario seeks to identify uniqueness in the records of the publicly available dataset.
**Journalist scenario**	If the adversary sets out to identify any individual from the publicly available dataset just to prove that it can be done by using another dataset for "matching" with the publicly available dataset, then we are under journalist re-identification risk scores.
**Secondary Research**	Consist of using already existing data for addressing questions out of scope for the original research which collected the data (primary research).

What are the UK researchers' views regarding their experiences with the de-identification, anonymisation, release methods and re-identification risk estimation for clinical trials datasets? A study protocol

Background

There is now a strong drive, particularly from publishers and funders, to encourage the release of relevant anonymised trial data sets [4]. Therefore, data-sharing has become an essential activity to disseminate current research, to enable new investigations and to maximise the scientific endeavour [5] [6] . Currently there are a number of such anonymised datasets made publicly available for secondary research via open or controlled access [7] [8]. Anonymisation of data is complex, and its implementation often means that the detail necessary to fully analyse the data is lost. There is therefore a balance between wanting to de-risk a dataset prior to sharing, against wanting it to be sufficiently detailed to answer valid research questions. So, we propose to explore the United Kingdom (UK) researchers' views regarding their experiences with the creation and release of de-identified/anonymised clinical trial datasets, the generation and use of re-identification risk scores, and their views about wider aspects of re-identification risks.

Why it is important to do this study?

We are currently investigating the re-identification risk scores across a range of clinical trials datasets [9]. Therefore, we want to better understand the views of UK researchers regarding their experiences with the creation and release of de-identified/anonymised clinical trial datasets, the generation and use of re-identification risk scores, and their views about wider aspects of re-identification risks. Knowing how other researchers are using such scores and in which context will help us identify how they could be useful in the future.

What are the UK researchers’ views regarding their experiences with the de-identification, anonymisation, release methods and re-identification risk estimation for clinical trials datasets? A study protocol

Objective

To explore clinical trials researchers’ views on their experiences with the creation and release of de-identified/anonymised clinical trial datasets, the generation and use of re-identification risk scores, and the wider aspects of re-identification risks.

Methods

Survey Design

The “checklist of questions for designing a survey study plan” by Creswell et at [10] was followed for the development of this protocol (see Appendix 1).

This study will use an online exploratory cross-sectional descriptive survey [10] [11] that consists of both open-ended and close-ended questions for data collection. This will allow us to gather information to better describe actual experiences regarding the investigated topic. The open-ended questions are especially important because of the lack of previous reporting on researchers’ views and experiences.

The survey will be in English. Most of the close-ended questions will have mutually exclusive choices, with a smaller number allowing for multiple answers [12] [13]. Where applicable, close-ended questions, will have an “other” (free text) option added to allow participants to provide an answer that is not available for selection [12]. Five-point response scales will be used for questions assessing frequency (always, often, sometimes, rarely, never).

The survey will be structured in five parts:

1. Consent and eligibility check.
2. Researchers’ work background details (current position, years of experience in current position and general place of work)

3 Researchers' experiences with the creation and release of de-identified/ anonymised clinical trial datasets

4. Researchers' awareness, knowledge and use regarding the generation of re-identification risk scores

5. Researchers' views about wider aspects of re-identification risks

The first draft of the survey is presented in Appendix 2 of this protocol.

The survey is designed to follow the layout presented in Figure 1.

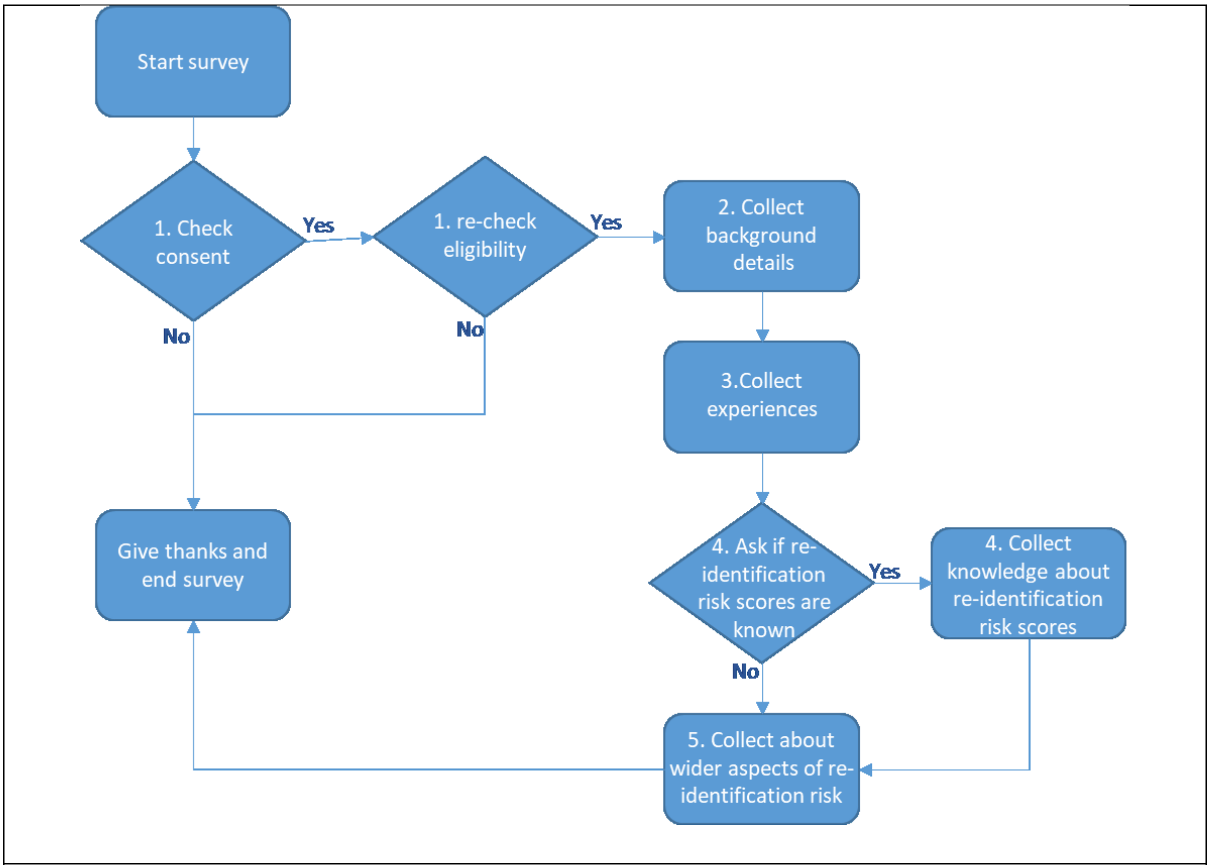

Figure 1. Single survey data collection process.

Therefore, a single participant (after the eligibility criteria has been met) will answer between 17 and 22 questions out of the proposed 24 questions, as some answers will determine the relevance of the next question.

The survey will be piloted with a selection of The University of Edinburgh personnel with experience in the processes of de-identification /anonymisation, release/maintenance and/or re-identification risk assessment of clinical trial datasets in order to prepare them for secondary research, before it is finalised and sent to the intended participants.

Study Population

Inclusion/Exclusion criteria: Clinical trial researchers based in the UK with experience in executing/overseeing any of the processes of de-identification /anonymisation, release/maintenance and/or re-identification risk assessment of clinical trial datasets in order to prepare them for secondary research.

Sampling and Recruitment

There will not be a formal sample size or stratification of the surveyed researchers as this is an exploratory study. Therefore, we will use convenience non-probability sampling[11] [14] [15] by providing a MS Forms[16] link or QR code with an introduction (email or print-out) (see Appendix 3) to:

- All 52 Clinical Trial Units (CTUs) registered in the UKCRC network (https://ukcrc-ctu.org.uk/registered-ctus/). We will email all UK fully/provisionally registered CTUs. We expect to obtain at least one survey per CTU. (population size n=52)
- The data transparency group at PHUSE (https://phuse.global/) (Contact via email, population size unknown).
- Allstat@JISCMAIL.AC.UK, an email discussion list for the UK Education and Research communities. (https://www.jiscmail.ac.uk/cgi-bin/webadmin?A0=allstat) (Contact via email, population size unknown).
- Participants at the 6th International Clinical Trials Methodology Conference (ICTMC) (3-6 October 2022) (Special event) (Contact via leaflet, Population size unknown).

The aim is to obtain as many responses as possible while the survey is active (around 5 weeks) to maximise the range of experiences. We estimate the population to be heterogeneous so a minimum of between 12-30 surveys is required[14] to reflect a wide range of views.

Data collection and extraction

This survey will not collect any personal data from the clinical trial researchers, and after extraction, all open questions will be carefully checked to make sure their coding do not contain any identifiable information. Only AR will be able to access all the data. We will use MS Forms as it provides the integrated web interface and data collection for the survey. The data by MS Forms "is encrypted both at rest and in transit" and it is stored in a European Server, all compliant with the General Data Protection Regulation (GPDR), for more detail please see:

https://support.microsoft.com/en-us/office/security-and-privacy-in-microsoft-forms-7e57f9ba-4aeb-4b1b-9e21-b75318532cd9

https://support.microsoft.com/en-us/office/data-storage-for-microsoft-forms-97a34e2e-98e1-4dc2-b6b4-7a8444cb1dc3

Analysis.

When the active period for the survey ends the response summary information and the individual responses of the complete surveys will be exported from MS Forms directly to AR's secured and password protected area at UoE or AR's DataStore allocation as per University of Edinburgh data handling policies. [17-19].

Individual responses will be kept until December 2023, then destroyed in accordance with University of Edinburgh policy for destroying archived research data (see https://www.ed.ac.uk/sites/default/files/atoms/files/dataprotectionhandbookv9.pdf and https://www.ed.ac.uk/data-protection/data-protection-policy).

Close-ended questions will be analysed using descriptive statistics (counts and percentages) on SAS 9.4 (or a more recent version). All this data will be analysed by AR and sense checked by another investigator (SCW, CJW, TJ).

Thematic analysis [20] [21] will be used to generate themes from the open-ended questions responded using NVivo® 12 (or a more recent version). All the data will be coded by AR and themes will be reviewed, defined and finalised in discussion with the multi-disciplinary research team to ensure valuable perspectives were included and help reduce subjectivity of findings (SCW, CJW, TJ).

This survey cannot investigate the response rate or the response bias to the survey. The results of this study will help to understand the views of UK researchers regarding their experiences with the creation and release of de-identified/anonymised clinical trial datasets, the generation and use of re-identification risk scores, and their views about wider aspects of re-identification risks.

Timetable

This is the proposed timetable for this study.

	Months					
	May2022	Jun2022	Sep2022	Oct2022	Nov2022	Dec2022
Ethics & Governance						
Protocol						
Pilot survey						
Main survey						
Analysis						
Report						

Potential sources of bias and limitations

This study is covering an emerging part of clinical trials research, for which even consensus about the definition of anonymisation does not exist [22]. This will create a variety in the views and experiences on de-identification / anonymisation of clinical trials datasets.

We acknowledge that this survey will potentially be filled out by highly motivated individuals with positives experiences, and we might not be fully engaging with researchers who have done de-identification / anonymisation but have had adverse experiences or difficulties in this area.

As this topic is very dense and our resources are limited, we are going to base the survey in the UK. Therefore it will not be possible to explore what is happening in other countries.

Ethics and dissemination

This project will not collect identifiable or personal participant data or personal sensitive information; therefore, this is a low risk project and we will follow the ethical review processes coordinated by the Edinburgh Medical School Research Ethics Committee (EMREC). Findings from this research will be presented at scientific conferences and

published in a peer-reviewed journal. No publication or presentation originating from this work will reveal any data that could lead to re-identification of individuals from the data collected.

Conflicts of Interests

The authors declare no competing interests.

Funding

AR has a scholarship from the University of Edinburgh to undertake a PhD with support from the Asthma UK Centre for Applied Research (AUKCAR). Neither funder (University of Edinburgh) nor sponsor (AUKCAR) contributed to protocol development.

CJW is supported in this work by NHS Lothian via the Edinburgh Clinical Trials Unit.

SCL is supported in this work by her employment at the Edinburgh Clinical Trials Unit.

TJ is supported by Asthma UK as part of the Asthma UK Centre for Applied Research (grant nos. AUK-AC-2012-01 and AUK-AC-2018-01),

SE is supported in this work by her employment at the Pragmatic Clinical Trials Unit.

Author contributions

AR, SCL and CJW conceived the idea for this work supported by SE. AR wrote the first draft, and all authors contributed to this article.

***Appendix 1** A checklist of Questions for Designing a Survey Study Plan (extracted from Chapter 8 in Research Design by John Creswell and J. David Creswell, 5th edition.) [10]*

Item ID	Item description	Protocol compliance
1	Is the purpose of the survey stated?	Yes
2	Are the reasons for choosing the design mentioned?	Yes
3	Is the nature of the survey (cross-sectional vs. longitudinal) identified	Yes
4	Is the population and its size mentioned?	Yes
5	Will the population be stratified? If so how?	Yes
6	How many people will be in the sample? On what basis was this size chosen?	Yes
7	What will be the procedure for sampling these individuals (e.g. random, non-random)?	Yes
8	What instrument will be used in the survey? Who developed the instrument?	Not applicable
9	What are the content areas addressed in the survey? The Scales	Yes
10	What procedure will be used to pilot or field test the survey?	Yes
11	What is the timeline for administering the survey?	Yes
12	What are the variables in the survey?	Yes
13	How do these variables cross-reference with the research questions and items on the survey?	Yes
14	What specific steps will be taken in data analysis to do the following	
14a	Analyse returns?	Yes
14b	Check for response bias?	Yes
14c	Conduct a descriptive analysis?	Yes
14d	Collapse items into scales?	Yes
14e	Check for reliability of scales?	Not applicable
14f	Run inferential statistics to answer research questions or assess practical implications of the results?	Not applicable
15	How will the results be interpreted?	Yes

Appendix 2 Survey (currently a separate file)

Appendix 3 Introduction email (currently a separate file)

Reference List

1. U.S. Government, *Health Insurance Portability and Accountability Act of 1996*, in *Public law*. 1996. p. 191.
2. U.S. Department of Health & Human Services (HHS), *Guidance Regarding Methods for De-identification of Protected Health Information in Accordance with the Health Insurance Portability and Accountability Act (HIPAA) Privacy Rule*. 2012.
3. Hrynaszkiewicz, I., et al., *Preparing raw clinical data for publication: guidance for journal editors, authors, and peer reviewers.* Trials, 2010. **11**(340).
4. Dal-Ré, R., *Access to Anonymized Individual Participant Clinical Trials Data: A Radical Change of Mind by the Most Prestigious Medical Journals.* Archivos de Bronconeumologia, 2018. **54**(2): p. 65-67.
5. Pisani, E., et al., *Beyond open data: realising the health benefits of sharing data.* BMJ, 2016. **355**: p. i5295.
6. Bertagnolli, M., et al., *Advantages of a truly open-access data-sharing model.* N Engl J Med, 2017. **12**(376): p. 1178-1181.
7. Clinical Study Data Request (CSDR). *Clinical Study Data Request*. Available from: https://clinicalstudydatarequest.com/.
8. The Yale University. *Yale University Open Data Access (YODA) Project*. [cited 2020 26 Oct 2020]; Available from: http://yoda.yale.edu/.
9. Rodriguez, A., et al., *What are the re-identification risk scores of publicly available anonymised clinical trial datasets? A study protocol* 2020, The University of Edinburgh p. 19.
10. Creswell, J.W. and J.D. Creswell, *Research design: Qualitative, quantitative, and mixed methods approaches*. 5th ed. 2018: Sage publications.
11. Fink, A., *The survey handbook*. 2003: sage.
12. Boynton, P.M. and T. Greenhalgh, *Selecting, designing, and developing your questionnaire.* Bmj, 2004. **328**(7451): p. 1312-1315.
13. Stehr-Green, P.A., et al., *Developing a questionnaire.* FOCUS Field Epidemiol, 2003. **2**(2): p. 1-6.
14. Dudovskiy, J., *The ultimate guide to writing a dissertation in business studies: A step-by-step assistance.* Pittsburgh, USA, 2016: p. 51.
15. Lavrakas, P.J., *Encyclopedia of survey research methods*. 2008, Thousand Oaks, California: Sage publications.
16. Microsoft, *MS Forms*. 2016. p. Part of Office 365.
17. The University of Edinburgh. *Data - Data Services*. 2020 [cited 2020 30 Oct 2020].
18. The University of Edinburgh. *Use University services*. 2020 [cited 2020 30 Oct 2020]; Available from: https://www.ed.ac.uk/infosec/information-protection-policies/procedures-guidance/use-university-services.
19. The University of Edinburgh. *Working with sensitive data* 2020 [cited 2020 30 Oct 2020]; Available from: https://www.ed.ac.uk/information-services/research-support/research-data-service/during/sensitive-data.
20. Braun, V. and V. Clarke, *Using thematic analysis in psychology.* Qualitative research in psychology, 2006. **3**(2): p. 77-101.
21. Gibbs, G.R., *Thematic coding and categorizing.* Analyzing qualitative data, 2007. **703**: p. 38-56.
22. Rodriguez, A., et al., *Current recommendations/practices for anonymising data from clinical trials in order to make it available for sharing: A scoping review*. 2022, The Univeristy of Edinburgh.
