# Supplementary material for: A survey on UK researchers’ views regarding their experiences with the de-identification, anonymisation, release methods and re-identification risk estimation for clinical trial datasets
Source: Clin Trials. 2024 Jun 19;22(1):11–23. doi: 10.1177/17407745241259086 (PMC11809122; doi:10.1177/17407745241259086)
Supplement: sj-pdf-3-ctj-10.1177_17407745241259086 – Supplemental material for A survey on UK researchers’ views regarding their experiences with the de-identification, anonymisation, release methods and re-identification risk estimation for clinical trial datasets [file sj-pdf-3-ctj-10.1177_17407745241259086.pdf]

An online survey of UK researchers' views on sharing anonymised clinical trials datasets.

Thank you for responding to this academic survey as part of a PhD at the Usher Institute at the University of Edinburgh. We would be grateful for your participation and value your views and experiences about de-identification, anonymisation, data release and re-identification risk estimation processes related to clinical trials datasets. The answers obtained will enhance the available evidence on this topic. Answering the survey questions should take no more than 15 minutes.

* Required

Participant Information about the survey

Introduction:
There are increasing pressures for anonymised datasets from clinical trials to be shared across the scientific community, However there is no a single standardised set of recommendations on how to anonymise and prepare clinical trial datasets for sharing. Therefore, this survey aims to explore the current views and experiences of researchers in the UK about de-identification/anonymisation, release methods and re-identification risk estimation processes for clinical trials datasets.

Study description:
This short questionnaire is arranged in four sections. The first section seeks information about your role. The second section collects your experience with the creation and release of de-identified/anonymised clinical trial datasets. The third section is about your views regarding the generation and use of re-identification risk scores. Finally, the fourth section is about your views about wider aspects of re-identification risks. **Text boxes in key questions allow you to add your comments, please do not leave them blank, if they are not applicable, just write NA**. The questions have been carefully selected and piloted to capture relevant views about the subject. The responses to most of the questions have been streamlined to require only a few clicks to select the answer. Answering the survey will take about 15 minutes to complete.

Risks and benefits:
Participation in this study is voluntary. You can withdraw from the study at any point before you have submitted your responses without giving a reason. The Edinburgh Medical School Research Ethics Committee (EMREC at The University of Edinburgh) has approved the survey (Approval Reference: **XXXXX**).
The responses to the survey will be anonymised at source and stored on a secured server provided by the University of Edinburgh. There are no benefits associated with your participation in this survey; however, this academic research could help to improve the knowledge about the existing practices on de-identification/anonymisation, data release and re-identification risk estimation.
Should the survey results be published, the anonymity of the responses will be not compromised. Further information about the Usher Institute's Privacy policy is available at https://www.ed.ac.uk/files/atoms/files/usher_institute_-_privacy_notice_v2.0_0.pdf (https://www.ed.ac.uk/files/atoms/files/usher_institute_-_privacy_notice_v2.0_0.pdf)

Eligibility and Invitation for participation:
We would appreciate your participation in this survey. Your opinion is very important to us. To proceed to the survey, please read the participant consent form and if satisfied, give your consent below.

1. Participant Consent Form

I agree to take part in this study and confirm the following:

- I have read the information provided above about this survey.
- I have had the opportunity to consider the information, which has been sufficient for giving my consent and I understand I will not receive any payment for taking part.
- I understand that my participation is voluntary and that I am free to withdraw at any time by closing the browser window without giving any reason and without my legal rights being affected. However, once I have pressed the submit button, I cannot withdraw my data as it is anonymous.
- I understand that the data collected during the study will be analysed by individuals the Usher Institute at the University of Edinburgh and regulatory authorities for audit purposes.
- I understand that taking part involves providing anonymous survey data and the responses may be shared with other researchers and used in future research projects, published in journals, and be used for teaching or academic material.
- By checking the box below, I confirm that I have understood and agree with the above statements, and I consent to taking part in this study.

*

○ I confirm I have read the above and give my consent.

Section 1. Background

For this survey:
De-identification refers to the removal of all personal health information and all other indirect identifiers which could lead to the identification of an individual. The most common de-identification methods are HIPPA (US Health Insurance Portability and Accountability Act of 1996) Safe harbour, in which 18 identifiers are removed from the datasets (https://www.hhs.gov/hipaa/for-professionals/privacy/special-topics/de-identification/index.html (https://www.hhs.gov/hipaa/for-professionals/privacy/special-topics/de-identification/index.html)) and Hrynaszkiewicz et al. (2010) (https://www.bmj.com/content/340/bmj.c181 (https://www.bmj.com/content/340/bmj.c181)) with an enhanced removal of potential identifiers which are commonly present in clinical trials datasets.
Anonymisation is when a dataset has been de-identified and then subsequent data manipulation/steps have been taken to further protect the dataset, for example, if a privacy model has been applied (e.g. k-anonymity) or the link with the original non anonymised dataset has been destroyed and this action cannot be reversed.
Secondary analysis: the use of existing de-identified/anonymised datasets, which were collected for a prior study, to investigate research questions outside the scope of the original study.

2. Eligibility. Are you involved or have you ever been involved with the de-identification/anonymisation of clinical trials dataset and/or with the processes for releasing de-identified/anonymised clinical trials dataset for secondary analysis? *

◯ Yes

◯ No

◯ Maybe

3. Are you based in the United Kingdom (UK)? *

◯ Yes

◯ No

4. Place of work. Are you currently affiliated to, or working at? *

◯ an UKCRC (Fully or provisionally) Registered Unit

◯ [ ] Other

5. What is your current job title or role? (Please choose the closest) *

◯ Director / Senior Manager

◯ Principal investigator

◯ Researcher (Research fellow/associate)

◯ Senior Researcher

◯ Statistician

◯ Senior Statistician

◯ Trial manager / coordinator

◯ Senior Trial manager / coordinator

◯ [ ] Other

6. Years of experience in that role *

- ◯ 0 to 2
- ◯ 3 to 5
- ◯ 6 or 10
- ◯ More than 10

Section 2. Your experiences on creation/release of de-identified/anonymised datasets

For this survey:
De-identification refers to the removal of all personal health information and all other indirect identifiers which could lead to the identification of an individual. The most common de-identification methods are HIPPA (US Health Insurance Portability and Accountability Act of 1996) Safe harbour, in which 18 identifiers are removed from the datasets (https://www.hhs.gov/hipaa/for-professionals/privacy/special-topics/de-identification/index.html (https://www.hhs.gov/hipaa/for-professionals/privacy/special-topics/de-identification/index.html)) and Hrynaszkiewicz et al. (2010) (https://www.bmj.com/content/340/bmj.c181 (https://www.bmj.com/content/340/bmj.c181)) with an enhanced removal of potential identifiers which are commonly present in clinical trials datasets.
Anonymisation is when a dataset has been de-identified and then subsequent data manipulation/steps have been taken to further protect the dataset, for example, if a privacy model has been applied (e.g. k-anonymity) or the link with the original non anonymised dataset has been destroyed and this action cannot be reversed.
Data release under controlled access: Datasets that can only be accessed if permission is granted by the data holders via their internal procedures.
Data release under open access: Datasets that can be accessed without any or minimal restrictions imposed by the data holders.

7. How are/were you involved with the de-identification/anonymisation of clinical trials dataset and/or with the processes for releasing de-identified/anonymised clinical trials dataset? (please tick all that apply) *

- ☐ Creation/Generation of de-identified/anonymised dataset
- ☐ Evaluation/Assessment/Peer review of de-identified/anonymised dataset
- ☐ Approval of the release of de-identified/anonymised dataset
- ☐ Generation/Evaluation/Assessment of de-identified/anonymised dataset re-identification risk
- ☐ Uploading/Maintenance/Distribution of de-identified/anonymised dataset
- ☐ ________ Other

8. Years of experience in dealing with de-identification/anonymisation and/or release of clinical trial datasets *

- ○ 0 to 2
- ○ 3 to 5
- ○ 6 or 10
- ○ More than 10

9. Which kind of documents do you currently use to assist you with the process to prepare and release de-identified/anonymised clinical trial datasets? *

- ○ Only internally developed documents/guidance (e.g. standardised operating procedures (SOPs), work instructions)
- ○ Only externally sourced documents/guidance (e.g. MRC guidance https://www.methodologyhubs.mrc.ac.uk/files/7114/3682/3831/Datasharingguidance2015.pdf (https://www.methodologyhubs.mrc.ac.uk/files/7114/3682/3831/Datasharingguidance2015.pdf), ICO anonymisation guidelines https://ico.org.uk/media/1061/anonymisation-code.pdf (https://ico.org.uk/media/1061/anonymisation-code.pdf))
- ○ Both internal and external documents/guidance
- ○ ________ Other

10. The internally developed documents/guidance (policies, SOPs or working instructions) at your organisation help to guide you with:

	Yes (documents/guidance implemented)	Yes (but document/guidance under construction)	No (this process is not covered)
The process of how to de-identify/anonymise clinical trials datasets	◯	◯	◯
The process for releasing de-identified/ anonymised clinical trials datasets	◯	◯	◯
The assessment of the re-identification risk of the de-identified/ anonymised clinical trials datasets.	◯	◯	◯

11. Which process do you mostly use for releasing de-identified/anonymised clinical trial data *

- ◯ Only de-identification, under controlled access
- ◯ De-identification followed by anonymisation, under controlled access
- ◯ Only de-identification, under open access
- ◯ De-identification followed by anonymisation, under open access
- ◯ Other

12. In your opinion, how was your experience in the process of **de-identifying/anonymising** clinical trials datasets? (e.g. what have worked? what have not worked? any concerns? any assurances?) *

13. In your opinion, how was your experience in the process of **releasing** de-identified/anonymised clinical trials datasets? (e.g. what have worked? what have not worked? any concerns? any assurances?) *

14. In your opinion, how was your experience in the process of **maintaining** released de-identified/anonymised clinical trials datasets? (e.g. what have worked? what have not worked? any concerns? any assurances?) *

Section 3. Your views about re-identification risks scores

For this survey:
Re-identification risk scores are defined as the estimated probabilities of any given individual being re-identified from an anonymised/de-identified dataset. The re-identification risk score depends on the variables available in the dataset, the number of observations in the dataset and on the strategy used to attack the dataset (prosecutor or journalist scenario).
Prosecutor scenario is when the adversary knows that a target individual (for whom identifiers are known) is in the publicly available dataset (released anonymised and/or de-identified).
Journalist scenario is when the adversary sets out to identify any individual from the publicly available dataset just to prove that it can be done by using another dataset for "matching" with the publicly available dataset.
(for more information view chapter 13 of El Emam, K. (2013). *Guide to the de-identification of personal health information*. CRC Press)

15. Are you aware of or ever come across re-identification risk scores for assisting in the release of de-identified/anonymised clinical trials datasets? *

- ◯ I have never heard of them
- ◯ I have heard about them, but I am not so sure what they are
- ◯ I have a general understanding, but do not use them
- ◯ I have a good understanding, and use them sometimes
- ◯ I have a strong understanding, and use them frequently

16. Which scenario(s) do you consider for calculating the re-identification risk score? *

- ◯ Prosecutor scenario (the adversary knows that a target individual is in the publicly available dataset)
- ◯ Journalist scenario (the adversary sets out to identify any individual from the publicly available dataset)
- ◯ Both
- ◯ Other

17. Please, describe how the calculated re-identification risk scores inform the process for data release. *

18. Which program do you use for calculating the re-identification risk scores? *

- ◯ SAS
- ◯ CRAN - Package sdcMicro
- ◯ Other

19. In your opinion, what are the advantages/disadvantages of using re-identification risk scores? *

20. How likely are you going to continue to use/generate the re-identification risk score? *

0	1	2	3	4	5	6	7	8	9	10

Not at all likely

Extremely likely

Section 3. Your views about re-identification risks score (Cont.)

For this survey:
Re-identification risk scores are the estimated probability of any given individual being re-identified from an anonymised/de-identified dataset. The re-identification risk score depends on the variables available in the dataset, the number of observations in the dataset and on the strategy used to attack the dataset (prosecutor or journalist scenario).

21. What are the barrier for not using the re-identification risk scores (please tick all that apply) *

- ☐ Lack of funding
- ☐ Lack of relevant training
- ☐ Lack of time
- ☐ ______

  Other

Section 4. Your views about wider aspects of re-identification risk

22. Each anonymised clinical trial dataset is unique, but in general terms, before any release of an anonymised dataset, do you think about: *

	Always	Often	Sometimes	Rarely	Never
Data format: number of participants, data standard/software used in datasets	○	○	○	○	○
Data Uniqueness: are there any unique values? Is there too much granularity in the datasets?	○	○	○	○	○
Sensitivity: the data still contains sensitivity information (e.g. stigmatising/rare disease, data about children/vulnerable individuals or family members)	○	○	○	○	○

23. Each anonymised clinical trial dataset is surrounded by a unique environment, but in general terms before any release of an anonymised dataset, do you think about: *

	Always	Often	Sometimes	Rarely	Never
Motivation: Existence of a motivated individual(s)/organisation(s) with resources and capabilities for an re-identification attack	○	○	○	○	○
Auxilliary information: Existence of matching datasets or other information sources to facilitate a re-identification attack	○	○	○	○	○
Geographical location: Most re-identification studies have been performed in countries with high internet penetration, arguably because there are more data available about individuals to make such attempts more likely to succeed.	○	○	○	○	○
Consequence: If a successful re-identification attack occurs, could it be in anyway harmful to individuals?	○	○	○	○	○
Consequence: If a successful re-identification attack occurs, could it be in anyway harmful to your organisation?	○	○	○	○	○

24. Please write below any other aspects you consider before you release de-identified /anonymised clinical trials data. *

This content is neither created nor endorsed by Microsoft. The data you submit will be sent to the form owner.

Microsoft Forms
