# Supplementary material for: A survey on UK researchers’ views regarding their experiences with the de-identification, anonymisation, release methods and re-identification risk estimation for clinical trial datasets
Source: Clin Trials. 2024 Jun 19;22(1):11–23. doi: 10.1177/17407745241259086 (PMC11809122; doi:10.1177/17407745241259086)
Supplement: sj-pdf-7-ctj-10.1177_17407745241259086 – Supplemental material for A survey on UK researchers’ views regarding their experiences with the de-identification, anonymisation, release methods and re-identification risk estimation for clinical trial datasets [file sj-pdf-7-ctj-10.1177_17407745241259086.pdf]

Additional file 6 List of registered CTUs (13NOV2022)

igniting our potential

UKCRC Registered Clinical Trials Units

2022/23 UKCRC Registration ID Numbers

ID	ORGANISATION	STATUS
32	Barts and the London Pragmatic CTU	FULL
4	Barts Clinical Trials Unit	FULL
1	Birmingham Clinical Trials Unit	FULL
70	Bristol Trials Centre*	FULL
3	CaCTUS (Cancer Clinical Trials Unit Scotland)	FULL
55	Cambridge Clinical Trials Unit (CCTU)	FULL
64	Cambridge Epidemiology & Trials Unit	FULL
6	Cancer Research UK Clinical Trials Unit (CRCTU)	FULL
7	Centre for Healthcare Randomised Trials (CHaRT)	FULL
63	Centre for Trials Research	FULL
56	Comprehensive CTU @ UCL	FULL
5	CR UK & UCL Cancer Trials Centre	FULL
14	Diabetes Trials Unit (Churchill Hospital, Oxford)	FULL
67	Derby Clinical Trials Support Unit (DCTSU)	FULL
15	Edinburgh Clinical Trials Unit, Edinburgh	FULL
65	Exeter Clinical Trials Unit	FULL
16	Glasgow Clinical Trials Unit	FULL
18	Imperial Clinical Trials Unit	FULL
42	Intensive Care National Audit & Research Centre (ICNARC) CTU	FULL
36	Keele Clinical Trials Unit	FULL
53	King's Clinical Trials Unit at King's Health Partners	FULL
41	Leeds Clinical Trials Research Unit	FULL
43	Leicester Clinical Trials Unit	FULL
12	Liverpool Trials Collaborative	FULL
44	London School of Hygiene & Tropical Medicine	FULL
9	Manchester Clinical Trials Unit	FULL
19	Medical Research Council Clinical Trials Unit at UCL	FULL
22	Newcastle Clinical Trials Unit (NCTU)	FULL
57	NHS Blood and Transplant Clinical Trials Unit	FULL
23	North Wales Organisation for Randomised Trials in Health (NWORTH)	FULL
25	Northern Ireland Clinical Trials Unit	FULL
51	Norwich Clinical Trials Unit	FULL
26	Nottingham Clinical Trials Unit	FULL
21	NPEU Clinical Trials Unit	FULL
46	Oxford Clinical Trial Service Unit & Epidemiological Studies Unit (CTSU)	FULL
27	Oxford Clinical Trials Research Unit (OCTRU)	FULL
52	Oxford Primary Care and Vaccines Collaborative Clinical Trials Unit	FULL
60	Papworth Trials Unit Collaboration	FULL
31	Peninsula Clinical Trials Unit	FULL
20	PRIMENT Clinical Trials Unit at UCL	FULL
62	Royal Marsden Clinical Trials Unit (RM-CTU)	FULL
34	Sheffield Clinical Trials Research Unit	FULL
37	Southampton Clinical Trials Unit	FULL
61	Surrey Clinical Trials Unit	FULL
58	Swansea Trials Unit	FULL
49	Tayside Clinical Trials Unit	FULL
17	The Institute of Cancer Research Clinical Trials & Statistics Unit (ICR- CTSU)	FULL

2022/23 UKCRC Registration ID Numbers

ID	ORGANISATION	STATUS
39	Warwick Clinical Trials Unit	FULL
40	York Trials Unit	FULL
68	Hull Health Trials Unit	PROV
69	Lancashire Clinical Trials Unit	PROV
66	Brighton and Sussex Clinical Trials Unit	PROV – UNDER REVIEW

*** Bristol Trials Centre is a merger of the Bristol Clinical Trials & Evaluation Unit (Reg ID 11) and the Bristol Randomised Trials Collaboration (Reg ID 2) both of which were fully registered units.**
