# Supplementary material for: A survey on UK researchers’ views regarding their experiences with the de-identification, anonymisation, release methods and re-identification risk estimation for clinical trial datasets
Source: Clin Trials. 2024 Jun 19;22(1):11–23. doi: 10.1177/17407745241259086 (PMC11809122; doi:10.1177/17407745241259086)
Supplement: sj-pdf-8-ctj-10.1177_17407745241259086 – Supplemental material for A survey on UK researchers’ views regarding their experiences with the de-identification, anonymisation, release methods and re-identification risk estimation for clinical trial datasets [file sj-pdf-8-ctj-10.1177_17407745241259086.pdf]

Additional file 7 favourable opinion on ethics

THE UNIVERSITY of EDINBURGH

Edinburgh Medical School
Research Ethics Committee (EMREC)

emrec@ed.ac.uk

Aryelly Rodriguez-Carbonell
Clinical Trials Statistician
Edinburgh Clinical Trials Unit

01 June 2022

Dear Aryelly

Study Title: What are the UK researchers' views regarding their experiences with the de-identification, anonymisation, release methods and re-identification risk estimation for clinical trials datasets

REC Reference: 22-EMREC-027

The Research Ethics Committee has now reviewed the above application.

Ethical opinion

The Committee can give a favourable ethical opinion of the above research on the basis described in the application form, protocol and supporting documentation, with no conditions.

In your EMREC form, the answer to E1 was missing; we have made the relevant amendment and saved with file name "EMREC Ethics Form v05 AIR_v 1.1 0106".

Amendments and Reporting Requirements

Now that you have a favourable ethical opinion from EMREC you are bound to the protocol, informed consent and data collection materials reviewed by us. Small changes like updating contact details, fixing typos, or adding a partner's logo do not require an amendment. However, you must re-contact us if you wish to make substantive changes that affect the protocol or answers to any of the questions on the EMREC form.

You should also contact EMREC to notify us about:

- Serious breaches of the protocol
- Safety reports and any adverse events

Favourable opinion from EMREC is not the only requirement to ensure integrity in research conduct, so in parallel with this stage, you will also want to satisfy yourself that you have considered other governance issues.

Documents reviewed

THE UNIVERSITY of EDINBURGH

The final list of documents reviewed and approved by the Committee is as follows:

**22-EMREC-027 Please quote this number on all correspondence**		
Document	*Version*	*Date*
EMREC Ethics Form	1.1	01/06/2022
Cover letter	1.0	04/05/2022
Protocol	1.0	28/04/2022
Survey (including information and consent form)	1.0	28/04/2022
Survey email	1.0	04/05/2022
ACCORD email (sponsorship not required)	1.0	29/04/2022
DPIA	1.0	03/05/2022
HRA tool results	1.0	28/04/2022
Data protection certificate	1.0	28/03/2020
DP for Research certificate	1.0	28/04/2022

With the Committee's best wishes for the success of this project.

Yours sincerely,

Sue Fletcher-Watson
Co-Chair, EMREC

Christine Campbell
Co-Chair, EMREC
